# Measures Determining Dementia-Related Attitudes in Adolescents: A Scoping Review

**DOI:** 10.1080/15350770.2023.2229837

**Published:** 2023-06-28

**Authors:** Esra Hassan, Ben Hicks, Naji Tabet, Nicolas Farina

**Affiliations:** a University of Sussex, Brighton, United Kingdom; b University of Plymouth, Plymouth, Devon, United Kingdom

**Keywords:** psychometric properties, validated measures, adolescents, scoping review, dementia attitudes

## Abstract

Reducing stigma is a key benefit to intergenerational programs. However, little is known about the availability and suitability of measures that capture dementia-related attitudes in adolescents, thus limiting interpretations of the efficacy of such programs. The aim of this scoping review was to provide an overview of outcome measures used to capture dementiarelated attitudes in adolescents. Scoping review methodology was used to systematically identify relevant articles. Key search terms included dementia, attitudes, and adolescents. Fourteen studies met the inclusion criteria, of which 13 unique measures were identified. However, there are gaps in psychometric properties and a lack of underlying theoretical frameworks.

## Introduction

Dementia has become an increasing public health priority in international policy. It is estimated that 55.2 million people currently live with dementia worldwide (Kafadar et al., 2021), with this number projected to increase further (GBD 2019 Dementia Forecasting Collaborators, 2022). Given the projected increase, the World Health Organization (WHO) outlined a need for greater dementia awareness in the general public as part of the wider goal to achieve a dementia-friendly society (WHO, 2017). Achieving a dementia-friendly society includes tackling some of the negative attitudes toward dementia held by the general public. Two thirds of the general public have misconceptions and a widespread fear of developing dementia (Alzheimer’s Disease International, 2019). Negative attitudes can have an impact on people living with dementia, their carers, and society at a psychological, economic, physical, and social level (Herrmann et al., 2018; Prince et al., 2016). For example, over 60% of people feel that it would be important to remove responsibilities from individuals living with dementia. Moreover, 20% of people stated that they would hide their dementia diagnosis from others (Alzheimer’s Disease International, 2019). These can lead to individuals socially withdrawing in order to conceal their dementia diagnosis from others and delay seeking help due to the fear of having their responsibilities, such as financial freedom, taken away from them.

Improving attitudes toward dementia may help tackle the barriers that individuals face in seeking timely diagnosis and care (Prince et al., 2016), consequently impacting quality of life for people living with dementia. Drawing upon the mental health literature, education about a group, protest against the inequity faced by a group, and contact with the stigmatized group are potential ways to help tackle negative attitudes (Mukadam & Livingston, 2012). Whilst initiatives such as the “Dementia Awareness Program” and intergenerational programs (Evans & Atkinson, 2017) attempt to address inaccurate stereotypes and beliefs (Chow et al., 2018), these are currently not based on rigorous empirical evidence. There is ambiguity as to the theoretical foundations used to inform such initiatives (Hebert & Scales, 2017) and the validity of dementia-related attitudes outcomes. These may undermine our understanding about what an effective initiative looks like.

A widely adopted definition of attitudes is that an attitude is an evaluation made on a particular object (or persons) through a psychological inclination that is expressed with some degree of liking or disliking (Eagly & Chaiken, 1993). Attitudes, alongside “stereotypes,” “perceptions,” “beliefs,” and “discrimination,” are all terms associated with stigma (Celious & Oyserman, 2001; Corrigan et al., 2005). While these terms represent distinct constructs, they all align with the central components of stigma frameworks. Stigma frameworks, such as the tripartite model, argue that attitudes are made up of three main components: cognitive (belief), affective (feelings and emotions), and behavioral responses (actions) (Eagly & Chaiken, 1995; Pryor & Reeder, 2011). These components are viewed as what is best captured by attitude measures (Eagly & Chaiken, 1993). A collection of negative attitudes and beliefs (cognitive responses) lead to discrimination and avoidance behaviors (behavioral responses) toward an attitudinal target (i.e., people with dementia) (Cheng et al., 2011).

There is limited literature on dementia related attitudes in adolescents in general despite theoretical frameworks highlighting the importance of the adolescent stage in attitude formation (e.g., “impressionable years hypothesis” (Krosnick & Alwin, 1989). Schools are also a potentially an optimal setting for delivering national and widespread initiatives since adolescents spend a large amount of their time at school. Schools can also reach adolescents from marginalized groups (Green et al., 2005) and aligns with citizenship education within the school curriculum. Targeting adolescents is important since they are an over-looked group in the general public regarding dementia-related attitudes research, with many studies focussing on attitudes held by healthcare professionals, carers, and adults over the age of 18 years old (Herrmann et al., 2018). This is despite evidence highlighting that adolescents do hold negative attitudes toward dementia and may develop negative attitudes at a young age (Farina, Griffiths, et al., 2020). There is a need to tackle attitudes at a generational level given that stereotypes are more susceptible to change in early adolescence and that adolescents appear more responsive to education and related initiatives (Werner et al., 2017). Intergenerational programs are one such way we can potentially improve attitudes and should be appropriately tailored to the relevant context and different communities, including adolescents (WHO, 2017). Therefore, we should have measures of dementia-related attitudes that are developed for this purpose, taking into consideration the unique characteristics and needs of adolescents.

Measures of dementia-related attitudes in adolescents are important to consider due to the lack of agreement on a “gold standard” measure for dementia-related attitudes in general (Harper et al., 2018). Measures not rigorously developed, may not accurately capture the underlying construct or lead to measurement error (Bound et al., 2001; Kimberlin & Winterstein, 2008). Even when a measure is rigorously developed for the general public, these cannot always be reliably used in a younger cohort (Isaac et al., 2017). Adolescents may have difficulties with the readability, complexity, and applicability of questionnaires compared to adults (i.e., 18 years and older) (Bell, 2007). As such, when intergenerational programs and other anti-stigma initiatives are developed for adolescents, it is unclear whether the measures used are accurately capturing attitudes in a standardized manner.

The main aim of this scoping review is to describe the outcome measures used when examining dementia-related attitudes in adolescents. To our knowledge, there are currently no reviews focusing on the measures used to assess dementia-related attitudes in adolescents. An overview of such measures will identify those that are validated and psychometrically sound, as well as the common pitfalls of these types of measures. This is important as it will provide a scope on further psychometric refinement before disseminating such measures widely (Farina, Hughes, et al., 2020; Parveen et al., 2020).

## Methods

Given the limited literature that exists in dementia-related attitudes in adolescents and the likelihood that there is a limited number of measures in this field, a scoping review was deemed more appropriate than a systematic review. Scoping reviews are useful for covering breadth of existing knowledge. The scoping review protocol is available upon request from the corresponding author.

The Arksey and O’Malley (2005) scoping review framework was adopted which consisted of the following steps: 1) identifying a research question, 2) identifying relevant studies, 3) study selection, 4) charting the data, and 5) collating and reporting the results (synthesis). In line with the framework, a risk of bias analysis was not conducted as it falls outside the scope of this review (Arksey & O’Malley, 2005). The framework was chosen for its transparency (Munn et al., 2018) and was amalgamated with additional scoping review recommendations by Levac et al. (2010) to further strengthen the process (e.g., defining concepts and target population for clearer direction on eligibility criteria) (Levac et al., 2010).

### Identifying the research question

The research question was as follows; “what measures are used to determine dementia attitudes and associated domains in adolescents?”

### Identifying relevant studies

Studies were identified through systematically searching electronic databases; PubMed, Web of Science, and PsycInfo (ProQuest). For literature inclusivity, no search filters, exclusionary Boolean operators or limitation on the time period were applied to the databases. A combination of MeSH terms, synonyms, variations in spelling for search words, text words, and Boolean operators were used to formulate the search string. Key search terms were in the English language and included those associated with “dementia” (dement* OR Alzheimer*), “stigma” (stigma OR perception OR attitude* OR discrimination OR “social distance” OR prejudice), and “adolescents” (adolescent* OR teen* OR “young people” OR child* OR student* OR “college students”). Final search strategies and date of searches were saved for replicability.

### Study selection

Only English language papers were included in the review. All articles were stored on the reference manager Zotero and underwent de-duplication. Two reviewers independently screened and examined the titles and abstracts on the Rayyan platform (Ouzzani et al., 2016), applying the eligibility criteria. At the full text screening stage, a similar independent review was conducted, and the level of agreement between the reviewers was determined using Cohen’s kappa coefficient (κ) for interrater reliability (a kappa result of 0.81–1.00 indicates almost perfect agreement) (McHugh, 2012). Disagreements on study inclusion were resolved between the two reviewers by having a discussion with a third independent reviewer who would be consulted should there have been difficulty in the reviewers coming to an agreement. Further relevant studies were identified from reference lists using the snowballing method and citation searches (“cited by”) (Pham et al., 2014).

### Eligibility

Inclusion criteria: a) English language papers, b) adolescents (10–18 years old), c) measures for dementia related stigma, d) quantitative outcomes, and e) peer-reviewed articles. Exclusion criteria: a) population targets above the age of 18 years old, b) populations of exclusively university students, c) qualitative methods and outcomes, d) studies reporting on specialist professions views exclusively (medical or healthcare professionals), e) measures exclusively measuring self-stigma or stigma by association, f) only measures with knowledge as an outcome, and g) gray literature (gray literature refers to information not produced by commercial publishers such as conference abstracts and blogs) (Adams et al., 2016).

While the WHO’s definition of adolescents includes individuals aged between 10 and 19 years (WHO, 2022), we have chosen to limit the inclusion to individuals up to 18 years old. This is to reflect standard education models. By excluding those aged 19 years old, potential heterogeneity introduced by including university students, who may have specialist training and are more likely to represent a biased cross-section of society (e.g., education level), is minimized. Studies were excluded if the average age of participants did not fall within the required age range (10–18 years old).

### Data charting and extraction

Data charting was performed on a pre-designed form for full text extraction. Extracted information included the following aspects: 1) study characteristics, including descriptive data such as study design and demographics, 2) psychometric properties where reported (validity and reliability), and 3) measure characteristics, such as a Likert scale and theoretical framework. Only directly reported and available data from the eligible studies were extracted.

### Data synthesis

The descriptive narrative for scoping reviews was adopted (Tricco et al., 2018). The data were categorized into themes based on the characteristics of the outcome measures such as validated measures used in the target population, validated measures used but not in the target population, and unvalidated measures. Validity and reliability outcomes indicated by p-values, α (alpha), and r (correlation coefficent were reported. The Preferred Reporting Items for Systematic Reviews and Meta-Analyses (PRISMA) study flow diagram was adopted for transparency of the selection, analysis, and reporting of the literature (Page & Moher, 2017).

## Results

**Figure 1:**
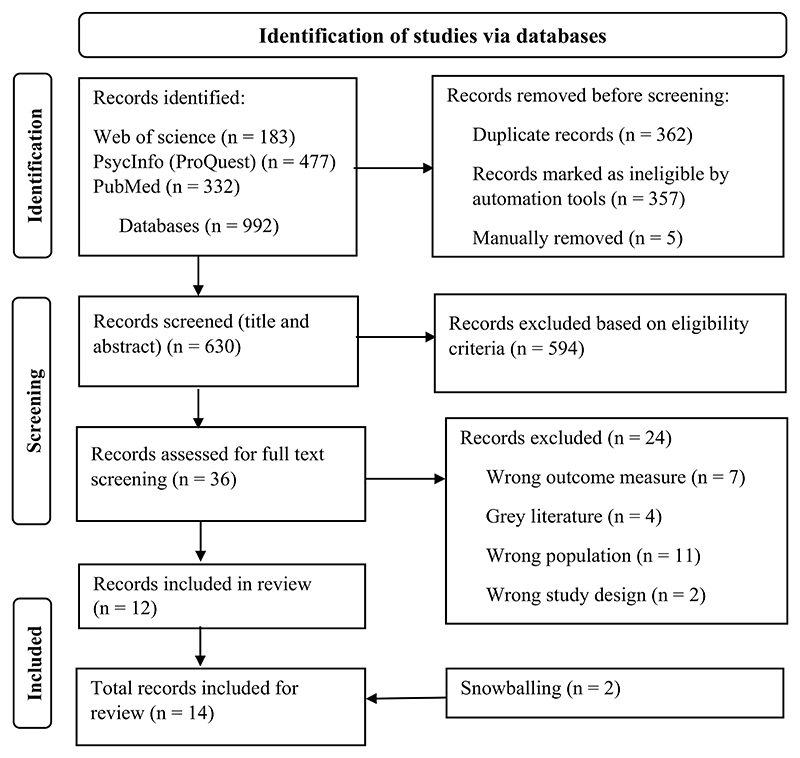
PRISMA study flow chart.

The dates of identified publications ranged from 1979 to 2022. The full text review had a 94.6% agreement rate (κ = 0.88) between the reviewers. As a result, 12 full-text articles were included in the review, and an additional two further studies identified through snowballing (checking reference lists of eligible studies) (Felc & Felc, 2020; Werner et al., 2017) were included. A total of 14 studies were extracted and synthesized. The PRISMA flow study diagram is presented in Figure 1.

### Study characteristics

Out of the 14 studies included in the review, nine studies had a quantitative study design, comprising of survey-based studies, two studies with a mixed-methods design and three studies with an intervention study design. Sample size ranged between four (Chow et al., 2018) and 5,515 (Fuh et al., 2005). The median sample size was 330. There were more females (58%) than males (42%) overall. Most of the studies did not report on ethnicity (10 studies). Where ethnicity was reported, participants were mostly homogenous (i.e., White British) (Farina, Hughes, et al., 2020, 2020; Griffiths et al., 2018). Three studies reported on nationality (Baker et al., 2018, 2019; Felc & Felc, 2020) with two studies recruiting Australians (>85%) and one study recruiting Slovenian participants. Studies were mostly conducted in England (k = 6) followed by Australia (k = 2) and Taiwan (k = 2) with one study each from the following: Canada, Israel, Macao, and Slovenia. The most common recruitment setting was schools. Specifically, three studies recruited from primary schools, while 11 studies recruited from secondary schools. Table 1 and supplementary material A provides an overview of the studies characteristics.

### What measures are used to determine dementia related-attitudes in adolescents?

Thirteen unique measures were identified, which were grouped into three categories: validated in target population, validated not in target population, and not validated. See supplementary material B.

### Validated measures in target population (<18-year-olds)

There were eight studies that used validated measures of dementia-related attitudes (Baker et al., 2018, 2019; Farina, Griffiths, et al., 2020; Farina, Hughes, et al., 2020, 2020; Griffiths et al., 2018; Lo et al., 2020; Werner et al., 2017). Five measures were identified: KIDS (Baker et al., 2018), the adapted version of the AQ-9 (Werner et al., 2017), the A-ADS (Griffiths et al., 2018), the brief A-ADS (Farina, Griffiths, et al., 2020), and the questionnaire of knowledge, attitude, and preventive practice of dementia care (Lo et al., 2020). Measure characteristics are outlined in Table 2.

### Theoretical frameworks

“Attitudes” was the most commonly measured construct with the outcome typically being “attitudes toward dementia” (KIDS, A-ADS, and brief A-ADS). Three of the measures adopted a theoretical framework. The AQ-9 (a shortened version of the AQ-27) adopted the attribution model of public discrimination (P. Corrigan et al., 2003; Werner et al., 2017), whilst the KIDS adopted the tripartite framework of attitudes (Baker et al., 2018). The brief A-ADS (Farina, Griffiths, et al., 2020) shares similarities with a public stigma framework (Rüsch et al., 2005), but it was not developed based on that framework.

### Psychometric properties

An overview of the psychometric properties of the validated measures is reported in Table 3.

### Validity

Construct validity was reported for the KIDS, A-ADS, and brief A-ADS measures. Exploratory factor analyses demonstrated a three-factor structure for both the KIDS (Baker et al., 2018) and the A-ADS measure (Farina, Griffiths, et al., 2020; Griffiths et al., 2018). The brief A-ADS demonstrated a single-factor structure (Farina, Griffiths, et al., 2020). Both the KIDS and A-ADS demonstrated convergent validity. The KIDS measure revealed a positive Pearson’s correlation with the DAS measure (*p* < .01), indicating that they measure similar constructs. Similarly, the A-ADS revealed a strong, positive correlation with attitudes toward older people scale (*p* < .001) (Griffiths et al., 2018), further suggesting the scales measure similar constructs. There was evidence of good concurrent validity for the KIDS and brief A-ADS with both significantly correlated with other measures within the dementia literature (Baker et al., 2018; Farina, Griffiths, et al., 2020; Farina, Hughes, et al., 2020), whilst a moderate, positive correlation between the brief A-ADS and KIDS was reported (Farina, Hughes, et al., 2020). Where content validity was reported, an item pooling procedure from the existing literature and feedback from an advisory committee was observed across the KIDS, A-ADS and questionnaire of knowledge, attitude, and preventive practice of dementia care. The brief A-ADS significantly was able to distinguish between self-reported positive attitudes toward dementia (*p* < .001) (Farina, Griffiths, et al., 2020). No study reported on criterion validity.

### Reliability

All measures were reported to have at least adequate reliability/internal consistency (>0.60), with most measures demonstrating good internal consistency (>0.70). Only one study explored test–retest reliability (Farina, Hughes, et al., 2020). The authors demonstrated that the brief A-ADS had acceptable test–retest reliability (*r* > 0.70) whilst the KIDS demonstrated “questionable reliability” (*r* < 0.50) (Farina, Hughes, et al., 2020). Inter-rater reliability was not reported on for any of the measures. See Table 3.

### Validated measures not in target population (>18-years old)

#### Measure characteristics

Five studies adopted measures that were validated in populations over 18-years-old. Three measures were identified from these studies: Allophilia scale (Kinney et al., 2017, used by; Farina, Hughes, et al., 2020; Farina, Griffiths, et al., 2020), the Dementia Attitudes Scale (DAS) (O’Connor & McFadden, 2010, used by; Baker et al., 2018; Griffiths et al., 2018; Liao et al., 2022), and the Young Adult Attitudes about Alzheimer’s disease Measure (Lundquist & Ready, 2008, used by; Griffiths et al., 2018). The latter two measures were validated in college (O’Connor & McFadden, 2010) and university students (Lundquist & Ready, 2008). Three studies used these measures for the purpose of developing or validating measures specifically designed for individuals under the age of 18 (Baker et al., 2018; Farina, Griffiths, et al., 2020; Griffiths et al., 2018). Four of the studies needed to simplify items of the Allophilia scale, DAS, and the Young Adult Attitudes about Alzheimer’s disease in order to make them more accessible to younger participants. For example, in the case of the DAS, “it is rewarding to work with people who have dementia” was reworded to “it is rewarding to play with people who have dementia” (Baker et al., 2018). In the study by Liao et al. (2022), the DAS was translated into Chinese for participants.

### Psychometric properties

#### Validity

The reported validity of these measures are presented in Table 3, where the correlations between these measures and the validated measures in individuals under the age of 18 are presented. Content, criterion and predictive validity for all three of the measures were not reported within the adolescent samples.

#### Reliability

The inter-rater reliability and test–retest reliability were not reported on for any of the measures when used in adolescents. Internal consistency was not reported for the Allophilia scale within the context of these studies on adolescents. The DAS had excellent internal consistency as indicated by Cronbach’s Alpha (α = 0.83) where previously validated (reported in Baker et al., 2018; Griffiths et al., 2018). This was supported by Baker et al. (2018) (*ωt* = .89) and Liao et al. (2022) (α = 0.85–0.87). Reliability for the young adult attitudes about Alzheimer’s disease was reported as “good” (cited in Griffiths et al., 2018).

### Unvalidated measures

A total of five studies used unvalidated measures of dementia-related attitudes in under 18-year-olds (Chow et al., 2018; Felc & Felc, 2020; Fox, 2020; Fuh et al., 2005; Isaac et al., 2017). There were no psychometric properties or theoretical framework underpinning them.

## Discussion

The findings of this scoping review reveal that there is limited literature measuring dementia-related attitudes in adolescents. However, out of 13 measures identified, it is encouraging that the majority of measures were validated within the target population. The brief A-ADS and KIDS were found to be the most used validated measures and had the most comprehensive psychometric properties, although this was in a small number of studies overall. To our knowledge, this scoping review is the first in the dementia-related attitudes literature to map out measures administered in adolescents using established scoping review methodology. In doing so, some of the limitations of the current literature base are available for researchers to consider and help with future work in capturing dementia-related attitudes in the adolescent demographic.

“Attitudes” was the most common construct measured amongst the studies. However, steps should be taken to reduce the ambiguity of what is being measured by defining the construct of interest. This is because the limitation of “Attitudes” is its variability in definition (Annear et al., 2015). Adopting a theoretical framework would help provide greater clarity regarding what is being measured. However, it is worth noting that there were only a limited number of measures (AQ-9 and KIDS) that adopted a theoretical framework, as mentioned by others in the past (Werner et al., 2020). It is important to note that the frameworks identified in this scoping review (attribution model of public discrimination and tripartite attitude framework) were developed with mental illness in mind (P. Corrigan et al., 2003; Eagly & Chaiken, 1993; Pryor & Reeder, 2011).

Internal consistency was reported for seven of the measures. Cronbach’s alpha is widely used to demonstrate reliability, with a coefficient alpha of 0.70 and above as the general rule of thumb for good reliability (Taber, 2018). Amongst the measures validated in the target population, there was a general indication for good internal consistency. The McDonalds Omega was used for the KIDS instead of Cronbach’s alpha (Baker et al., 2018) which some meth-odologists argue is a more optimal measure of reliability for unidimensional constructs (Goodboy & Martin, 2020; Hayes & Coutts, 2020). Test–retest reliability was not commonly reported across the measures, although this type of stability testing is not necessarily deemed the most appropriate for constructs that are expected to change over time such as attitudes (De Von et al., 2007). This may explain the “questionable” test–retest reliability score reported on the KIDS and “acceptable” reliability for the brief A-ADS (Farina, Hughes, et al., 2020).

Almost all of the measures that were validated in populations over 18-year-olds required adjustments to item wording to make them more accessible to the younger participants (e.g., Baker et al., 2018). Adapting/removing items may alter the psychometric outcomes of the original measure and therefore measures with these modifications should undergo further psychometric testing to ensure they are fit for purpose within a younger demographic. Only one study reported on measure readability (Farina, Griffiths, et al., 2020). This is a notable limitation common in child measures but are important to the ease and understanding of text for children (Oakland & Lane, 2004; Patalay et al., 2018) due to cognitive effort differences between adults and children (Bell, 2007; Krosnick & Alwin, 1989). These pitfalls are important to consider since these have implications on accurately capturing attitudes in intergenerational programs. Accurately measuring attitudes in both older adults and younger people, such as adolescents, is vital in understanding the benefits of inter-generational programs. This is particularly important when comparing the effectiveness of various intergenerational programs that aim to tackle negative dementia attitudes (Farina, Griffiths, et al., 2020; Silverstein & Sherman, 2010). There are several limitations to this scoping review. First, there were relatively few studies that met this review’s inclusion criteria. Whilst this highlights a need for further work in this area of the literature, we are limited as to how confidently we can apply these recommendations to other countries. Second, the psychometric properties of questionnaires were limited to those that were reported within the included studies. As such, some of conclusions may be limited by the quality of reporting. Lastly, this scoping review did not implement all the recommendations by Levac et al. (2010). Implementing the optional sixth stage (consulting) (Arksey & O’Malley, 2005) would have added methodological rigor and further sources of information to the review (Levac et al., 2010). However, there is no widely accepted consensus on how to approach consultation in scoping reviews. A review on scoping review methodology found that scoping reviews rarely report consultation exercises in meaningful detail. This may be attributed to power imbalances between researchers and stakeholder consultants, as well as ethical implications regarding whether stakeholder consulting is participatory research in itself (Buus et al., 2022). The consultation exercise was therefore considered beyond the scope of this review.

## Conclusion

Whilst just over half of the studies exploring dementia-related attitudes in adolescents used validated measures, there is still a clear gap in terms of psychometric properties reported and the underlying theoretical framework. Measures that have been validated in other populations should take precedence over unvalidated measures since these types of measures have no psychometric support. To date, the brief A-ADS and KIDS have the most robust evidence of psychometric validity for measuring dementia related-attitudes in adolescents.

## Figures and Tables

**Table 1:** Study characteristics of studies measuring dementia-related stigma and associated domains.

Author and year of publication	Study design	Country	Recruitment setting	Name of measures used	Outcomes
Baker et al., (2019)	Mixed methods Design	Australia	Three schools	Kids Insight into Dementia Survey (KIDS)	Improved attitudes toward people with dementia for students who participated in Kids4Dementia.
Baker et al., (2018)	Quantitative – scale development	Australia	Three schools	KIDS; Dementia attitudes scale (DAS)	KIDS 52 item reduced to 14 items and three-factor solution identified.
Chow et al., (2018)	Intervention	Canada	NR	Survey developed for the program to evaluate attitudes. Assessment of students’ perception	Students expressed significantly more positive attitudes following intervention.
Farina et al., (2020)	Quantitative – questionnaire	England	Four schools	Adolescent attitudes toward Dementia scale (A-ADS); Allophilia scale	More adolescents had positive or neutral attitudes toward dementia whilst a proportion of adolescents had misconceptions or negative attitudes.
Farina et al., (2020)	Quantitative – scale development	England	NR	Brief Adolescent attitudes toward Dementia scale (Brief A-ADS); Allophilia scale; A-ADS	Brief A-ADS had good internal consistency, predictive and concurrent validity.
Farina et al., (2020)	Intervention	England	Three schools	Brief A-ADS; KIDS	Dementia Friends is successful in reach and impact but may fall short on improving attitudes toward dementia.
Felc and Felc, (2020)	Quantitative – questionnaire	Slovenia	Targeted 50 schools	Questionnaire (name not stated)	Adolescent students had positive attitudes toward activities for reducing dementia risk. Some responses to items reflected more negative attitudes.
Fox, (2020)	Mixed methods design	England	Two schools	Card selection task - name not stated	Depression, anorexia nervosa and dementia elicit differing responses in children.
Fuh et al., (2005)	Quantitative – questionnaire	Taiwan	Seven schools	Attitude toward Dementia Questionnaire	Most children and adolescents had overly optimistic attitude toward dementia.
Griffiths et al., (2018)	Quantitative – scale development	England	Four schools	A-ADS; DAS; Young adult attitudes about Alzheimer’s disease measure	A-ADS captures three factors: perceptions of dementia, personal sacrifice, and empathy with people with dementia.
Isaac et al., (2017)	Quantitative – questionnaire	England	Two schools	No name given	Adolescent students had both positive and negative attitudes toward dementia.
(Liao et al., 2022)	Intervention	Taiwan	Nine schools	DAS	Exergaming improved attitudes of adolescents toward dementia and older adults.
Lo et al., (2020)	Quantitative – questionnaire	Macao	Ten schools	Knowledge, attitude and preventive practice of dementia care	Positive relationship between preventive practice and attitude and knowledge.
Werner et al., (2017)	Quantitative – questionnaire	Israel	Two schools	Adapted version of the Attribution Questionnaire 9 (AQ-9)	Higher levels of stigma toward a person with Alzheimer’s disease in Israeli Arab students compared to Jewish students.

NR= Not Reported. KIDS (Kids Insight into Dementia Survey); DAS (dementia attitude scale); A-ADS (adolescent attitudes toward dementia scale); Brief A-ADS (Brief adolescent attitudes toward dementia scale); AQ-9 (Attribution Questionnaire 9).

**Table 2:** Measure characteristics of validated measures in adolescents and children.

Validated measures	Developed by	Eligible studies using the measure	Outcome of measure	Theoretical framework	Geographic location (number of studies)
Kids Insight into Dementia Survey (KIDS)	Baker et al., (2018)	3	Knowledge and dementia attitudes	Tripartite framework of attitudes	Australia (2) England (1) England (1)
Adapted version of the Attribution Questionnaire-27 (AQ-9)	Corrigan et al., (2003)	1	Public stigma toward a person with Alzheimer’s Disease	Attribution model of public discrimination toward mental illness (P. Corrigan et al., 2003)	Israel (1)
The Adolescent Attitudes toward Dementia Scale (A-ADS)	Griffiths et al., (2018) based on the DAS (O’Connor and McFadden, 2010) and the Young adult attitudes about Alzheimer’s disease measure (Lundquist and Ready, 2008)	3	Adolescent attitudes toward dementia	NR	England (3)
The Brief Adolescent Attitudes toward Dementia Scale (Brief A-ADS)	Farina et al., (2020) based on the 23-item version of A-ADS (Griffiths et al., 2018)	2	Adolescent attitudes toward dementia	Public stigma framework (Rüsch et al., 2005)	England (2)
Questionnaire of knowledge, attitude and preventive practice of dementia care	Chi et al., (2017) and Yang et al., (2015)	1	Knowledge and attitudes of dementia care	NR	Macao (1)

NR = Not Reported.

**Table 3:** Psychometric characteristics of the validated measures identified in eligible studies (<18-years-old).

Psychometric properties	Validated Measures in target population (10-18years old) summary				
	KIDS (Baker et al., 2018)	AQ-9 (Werner et al., 2017)	A-ADS 2017) (Griffiths et al., 2018)	Brief A-ADS (Farina, Griffiths, et al., 2020)	Questionnaire of knowledge, attitude and preventive practice of dementia care (Lo et al., 2020)
Construct validity	Good construct validity. Of 33 items, 14 items retained. EFA shows three factor solution (personhood, stigma and dementia understanding) (Baker et al., 2018).	NR	EFA: scale captures three factors (perceptions of dementia, personal sacrifice, and empathy with people living with dementia) (Farina, Griffiths, et al., 2020; Griffiths et al., 2018). Good underlying construct. From 30 items, 23 items were selected to form the A-ADS. Scale designed to yield a single score reflecting one underlying construct of “attitudes toward dementia” (Griffiths et al., 2018).	EFA demonstrates a single factor structure (perceptions) for the 13-item. 23 items reduced to 13 items scale (Farina, Griffiths, et al., 2020).	NR
Convergent validity	Strong positive Pearson’s correlation between KIDS and DAS (r= 0.76, p < .01) (Baker et al., 2018)	NR	A-ADS and young adult attitudes about Alzheimer’s disease strongly correlated (r= .94, *p *< .001) (Griffiths et al., 2018). Strong Pearson’s correlation also between A-ADS and DAS (r= .75, *p <* .001) (Griffiths et al., 2018)	NR	NR
Content validity	Item pool of cognitive, affective and behavioral intention items from two sources. Advisory committee (n = 6) reached consensus on master list of 52 items (Baker et al., 2018).	NR	A-ADS developed based on items from the DAS and Lundquist and Ready scale. 15 cognitive interviews conducted with young people aged 14-17 years old. Based on feedback, draft of 30 items for A-ADS (Griffiths et al., 2018).	NR	The 30-item questionnaire developed based on questionnaires from Chinese communities, Chi et al. (2017) and Yang et al. (2015). Validated by 5 experts. The Content Validity Index =0.973 (Lo et al., 2020).
Criterion validity	NR	NR	NR	NR	NR
Predictive validity	NR	NR	NR	EFA: 13 item A-ADS has good predictive validity (t = -5.53, *p *< .001 ). CFA shows 13 item A-ADS had good predictive validity (t = -6.01, *p <* .001 ) (Farina, Griffiths, et al., 2020).	NR
Concurrent validity	14-item KIDS and DAS (r= .76, *p* <.01) (Baker et al., 2018). KIDS and children’s social desirability scale had weak positive correlation (r= .20, p < .05) (Baker et al., 2018).	NR	NR	EFA: A-ADS 13 item good concurrent validity with Allophilia scale (r = 0.77, p<.001). CFA: good concurrent validity with Allophilia scale (r = 0.73, *p* < .001 ). 23 item A-ADS and 13 item A-ADS very strong positive association (r = 0.95, *p <* .001) (Farina, Griffiths, et al., 2020; Farina, Hughes, et al., 2020). Brief A-ADS and KIDS had moderate positive association with each other (r = 0.47—0.67) (Farina, Hughes, et al., 2020).	NR
Inter-rater reliability	NR	NR	NR	NR	NR
Test-Retest Reliability	“Questionable reliability” (r = 0.55, *p <* .0001) (Farina, Hughes, et al., 2020).	NR	NR	Pearson’s correlation shows “acceptable reliability” (r= 0.78, p < .0001) (Farina, Hughes, et al., 2020).	NR
Internal consistency	Good reliability (Farina, Hughes, et al., 2020;). The McDonald’s Omega indicated a good internal consistency (*ωt* = .83) for 14 item KIDS (Baker et al., 2018).	Good internal reliability was found (r = .29, .48, and .29 for the cognitive, emotional, and behavioral dimensions, respectively, *p *<.0001) (Werner et al., 2017).	The three sub scales showed adequate internal consistency: personal sacrifice sub scale (α = .79), empathy with people with dementia (α = .69), perceptions of dementia (α= .61) (Griffiths et al., 2018). Good reliability (α = 0.85) (Farina, Griffiths, et al., 2020).	EFA: brief A-ADS had good internal consistency (a = 0.88). CFA: 13-item A-ADS had high internal consistency (α = 0.83) (Farina, Griffiths, et al., 2020).	Cronbach alpha *r =* 0.808 (Lo et al., 2020)

NR= Not Reported; CFA = confirmatory factor analysis; EFA = exploratory factor analysis; KIDS (Kids Insight into Dementia Survey); DAS (dementia attitude scale); A-ADS (adolescent attitudes toward dementia scale); Brief A-ADS (Brief adolescent attitudes toward dementia scale).

## References

[R1] Adams J, Hillier-Brown FC, Moore HJ, Lake AA, Araujo-Soares V, White M, Summerbell C (2016). Searching and synthesising ‘grey literature’ and ‘grey information’ in public health: Critical reflections on three case studies. Systematic Reviews.

[R2] Alzheimer’s Disease International (2019). World Alzheimer report 2019: Attitudes to Dementia.

[R3] Annear MJ, Toye C, McInerney F, Eccleston C, Tranter B, Elliott KE, Robinson A (2015). What should we know about dementia in the 21st century? A Delphi consensus study. BMC Geriatrics.

[R4] Arksey H, O’Malley L (2005). Scoping studies: Towards a methodological framework. International Journal of Social Research Methodology.

[R5] Baker JR, Goodenough B, Jeon Y-H, Bryden C, Hutchinson K, Low L-F (2019). The Kids4Dementia education program is effective in improving children’s attitudes towards dementia. Dementia.

[R6] Baker JR, Low LF, Goodenough B, Jeon YH, Tsang RS, Bryden C, Hutchinson K (2018). The Kids Insight into Dementia Survey (KIDS): Development and preliminary psychometric properties. Aging & Mental Health.

[R7] Bell A (2007). Designing and testing questionnaires for children. Journal of Research in Nursing.

[R8] Bound J, Brown C, Mathiowetz N (2001). Handbook of econometrics.

[R9] Buus N, Nygaard L, Berring LL, Hybholt L, Kamionka SL, Rossen CB, Juel A (2022). Arksey and O′ Malley’s consultation exercise in scoping reviews: A critical review. Journal of Advanced Nursing.

[R10] Celious A, Oyserman D (2001). Race from the inside: An emerging heterogeneous race model. Journal of Social Issues.

[R11] Cheng ST, Lam LC, Chan LC, Law AC, Fung AW, Chan WC, Chan WM (2011). The effects of exposure to scenarios about dementia on stigma and attitudes toward dementia care in a Chinese community. International Psychogeriatrics.

[R12] Chi YC, Liu MF, Hsiao YL (2017). A study on nursing assistants’ knowledge and attitude of dementia care. The Journal of Long-Term Care.

[R13] Chow S, Chow R, Yu C, Nadalini O, Krcmar D, DeAngelis C, Herrmann N (2018). Dementia awareness for high school students: A pilot program. International Public Health Journal.

[R14] Corrigan PW, Kerr A, Knudsen L (2005). The stigma of mental illness: Explanatory models and methods for change. Applied and Preventive Psychology.

[R15] Corrigan P, Markowitz FE, Watson A, Rowan D, Kubiak MA (2003). An attribution model of public discrimination towards persons with mental illness. Journal of Health and Social Behavior.

[R16] De Von HA, Block ME, Moyle-Wright P, Ernst DM, Hayden SJ, Lazzara DJ, Savoy SM, Kostas-Polston E (2007). A psychometric toolbox for testing validity and reliability. Journal of Nursing Scholarship.

[R17] Eagly AH, Chaiken S (1993). The psychology of attitudes.

[R18] Eagly AH, Chaiken S (1995). Attitude strength, attitude structure, and resistance to change. Attitude Strength: Antecedents and Consequences.

[R19] Evans S, Atkinson T (2017). An intergenerational approach to addressing stigma. Innovation in Aging.

[R20] Farina N, Griffiths AW, Hughes LJ, Parveen S (2020). Measuring adolescent attitudes towards dementia: The revalidation and refinement of the A-ADS. Journal of Health Psychology.

[R21] Farina N, Hughes LJ, Griffiths AW, Parveen S (2020). Adolescents’ experiences and perceptions of dementia. Aging & Mental Health.

[R22] Farina N, Hughes LJ, Jones E, Parveen S, Griffiths AW, Galvin K, Banerjee S (2020). The effect of a dementia awareness class on changing dementia attitudes in adolescents. BMC Geriatrics.

[R23] Felc Z, Felc B (2020). Knowledge of modifiable dementia risk factors among Slovenian adolescents. International Journal of Psychiatry Research.

[R24] Fox C (2020). Children’s attitudes to people with mental illness. Journal of Applied Developmental Psychology.

[R25] Fuh J-L, Wang S-J, Juang K-D (2005). Understanding of senile dementia by children and adolescents: Why grandma can’t remember me?. Acta Neurol Taiwan.

[R26] GBD 2019 Dementia Forecasting Collaborators (2022). Estimation of the global prevalence of dementia in 2019 and forecasted prevalence in 2050: An analysis for the global burden of disease study 2019. The Lancet Public Health.

[R27] Goodboy AK, Martin MM (2020). Omega over alpha for reliability estimation of unidimensional communication measures. Annals of the International Communication Association.

[R28] Green J, Howes F, Waters E, Maher E, Oberklaid F (2005). Promoting the social and emotional health of primary school-aged children: Reviewing the evidence base for school-based interventions. International Journal of Mental Health Promotion.

[R29] Griffiths AW, Parveen S, Shafiq S, Oyebode JR (2018). Development of the Adolescent Attitudes towards Dementia Scale (A‐ADS). International Journal of Geriatric Psychiatry.

[R30] Harper LA, Dobbs BM, Buckwalter K (2018). Stigma in dementia: Its time to talk about it. Innovation in Aging.

[R31] Hayes AF, Coutts JJ (2020). Use omega rather than cronbach’s alpha for estimating reliability. But . . .. Communication Methods and Measures.

[R32] Hebert CA, Scales K (2017). Dementia friendly initiatives: A state of the science review. Dementia.

[R33] Herrmann LK, Welter E, Leverenz J, Lerner AJ, Udelson N, Kanetsky C, Sajatovic M (2018). A systematic review of dementia-related stigma research: Can we move the stigma dial?. The American Journal of Geriatric Psychiatry.

[R34] Isaac MG, Isaac MM, Farina N, Tabet N (2017). Knowledge and attitudes towards dementia in adolescent students. Journal of Mental Health.

[R35] Kafadar AH, Barrett C, Cheung KL (2021). Knowledge and perceptions of Alzheimer’s disease in three ethnic groups of younger adults in the United Kingdom. BMC Public Health.

[R36] Kimberlin CL, Winterstein AG (2008). Validity and reliability of measurement instruments used in research. American Journal of Health-System Pharmacy.

[R37] Kinney JM, Yamashita T, Brown JS (2017). Measuring positive attitudes toward persons with dementia: A validation of the Allophilia scale. Dementia.

[R38] Krosnick JA, Alwin DF (1989). Aging and susceptibility to attitude change. Journal of Personality and Social Psychology.

[R39] Levac D, Colquhoun H, O’Brien KK (2010). Scoping studies: Advancing the methodology. Implementation Science.

[R40] Liao YJ, Lin LC, Wu SC, Fuh JL, Chiang I, Gau BS (2022). Comparison of long-term effects of exergaming (Xbox one kinet) and companionship programs on attitude towards dementia and the older adults among adolescents: A quasi-experimental longitudinal study. BMC Geriatrics.

[R41] Lo IL, Zeng W, Lei CI, Lam C, Lou HL (2020). High school students’ knowledge, attitude and preventive practice of dementia care in macao. American Journal of Alzheimer’s Disease & Other Dementias®.

[R42] Lundquist TS, Ready RE (2008). Young adult attitudes about alzheimer’s disease. American Journal of Alzheimer’s Disease & Other Dementias®.

[R43] McHugh ML (2012). Interrater reliability: The kappa statistic. Biochemia Medica.

[R44] Mukadam N, Livingston G (2012). Reducing the stigma associated with dementia: Approaches and goals. Aging Health.

[R45] Munn Z, Peters MDJ, Stern C, Tufanaru C, McArthur A, Aromataris E (2018). Systematic review or scoping review? Guidance for authors when choosing between a systematic or scoping review approach. BMC Medical Research Methodology.

[R46] Oakland T, Lane HB (2004). Language, reading, and readability formulas: Implications for developing and adapting tests. International Journal of Testing.

[R47] O’Connor ML, McFadden SH (2010). Development and psychometric validation of the dementia attitudes scale. International Journal of Alzheimer’s Disease.

[R48] Ouzzani M, Hammady H, Fedorowicz Z, Elmagarmid A (2016). Rayyan—A web and mobile app for systematic reviews. Systematic Reviews.

[R49] Page MJ, Moher D (2017). Evaluations of the uptake and impact of the Preferred Reporting Items for Systematic reviews and Meta-Analyses (PRISMA) statement and extensions: A scoping review. Systematic Reviews.

[R50] Parveen S, Griffiths AW, Farina N (2020). The development and validation of the adolescent level of contact with dementia scale. International Journal of Geriatric Psychiatry.

[R51] Patalay P, Hayes D, Wolpert M (2018). Assessing the readability of the self-reported strengths and difficulties questionnaire. BJPsych Open.

[R52] Pham MT, Rajić A, Greig JD, Sargeant JM, Papadopoulos A, McEwen SA (2014). A scoping review of scoping reviews: Advancing the approach and enhancing the consistency. Research Synthesis Methods.

[R53] Prince M, Ali G-C, Guerchet M, Prina AM, Albanese E, Wu Y-T (2016). Recent global trends in the prevalence and incidence of dementia, and survival with dementia. Alzheimer’s Research & Therapy.

[R54] Pryor J, Reeder G, Hall B, Hall J, Cockerell C (2011). HIV/AIDS in the Post-HAART Era: Manifestations, Treatment and Epidemiology.

[R55] Rüsch N, Angermeyer MC, Corrigan PW (2005). Mental illness stigma: Concepts, consequences, and initiatives to reduce stigma. European Psychiatry.

[R56] Silverstein NM, Sherman R (2010). Taking control of Alzheimer’s disease: A training evaluation. Gerontology & Geriatrics Education.

[R57] Taber KS (2018). The use of Cronbach’s alpha when developing and reporting research instruments in science education. Research in Science Education.

[R58] Tricco AC, Lillie E, Zarin W, O’Brien KK, Colquhoun H, Levac D, Straus SE (2018). PRISMA extension for scoping reviews (PRISMA-ScR): Checklist and explanation. Annals of Internal Medicine.

[R59] Werner P, Jabel HA, Reuveni Y, Prilutzki D (2017). Stigmatic beliefs toward a person with Alzheimer’s disease among high-school students: Does majority–minority status make a difference?. Educational Gerontology.

[R60] Werner P, Raviv-Turgeman L, Corrigan PW (2020). The influence of the age of dementia onset on college students’ stigmatic attributions towards a person with dementia. BMC Geriatrics.

[R61] World Health Organization Adolescent Health.

[R62] World Health Organization (2017). Global action plan on the public health response to dementia.

[R63] Yang H, Yang G, Cheng M, Cong J (2015). Survey on the status and its correlation of knowledge, attitude and behaviors related to Alzheimer’s disease among community residents in Tianjin. Chinese Journal of Practical Nursing.

